# Immediate and conventional loading of anterior dental implants: Aesthetic considerations and outcomes

**DOI:** 10.6026/973206300200980

**Published:** 2024-09-30

**Authors:** Aravind Anto, Sunil Sharma, Amit Kumar Sharma, Vikram Sharma, Harshita Bisharwal, Rachana Sharma

**Affiliations:** 1 Department of oral and maxillofacial surgery, Nims dental college and Hospital, Nims University, Jaipur, India

**Keywords:** Immediate loading protocol, conventional loading protocol, dental implants, peri- implant bone loss

## Abstract

Dental implants, made from inert materials, integrate into bone to replace missing teeth. Recent years have seen scrutiny in modifying the traditional approach with early and immediate loading of implants in various clinical contexts. This study emphasizes in evaluating the parallelism between immediately and conventionally loaded dental implants following implant placement using peri- implant bone loss in anterior region. A randomized control trial was done on 30 patients having missing anterior teeth. Patient in group A were treated with immediate loading protocol and patient in group B were treated with conventional loading protocol. Patients were assessed postoperatively at 6 and 12 months for peri-implant bone level changes, plaque score, gingival score, calculus score, and papilla preservation score. Intergroup comparisons of marginal bone loss on the mesial side between group A and B at 6 months 0.17 and 0.21 and 12 months 0.30 and 0.34 were not statistically significant (p > 0.05). Similarly, marginal bone loss on the distal side at 6-months is 0.20 and 0.32 and 12-months are 0.44 and 0.34 showed no statistical significance (p > 0.05). Intergroup comparisons of plaque index (PI), gingival index (GI), calculus index (CI), and papilla preservation index (PPI) also indicated no significant differences across different time intervals. Therefore, data concludes that both immediate loading and conventional loading showed no significant difference proving immediate loading protocol is as effective as conventional loading and thus it can be effectively applied in case where patients' aesthetics are a main concern.

## Background:

Dentition is a vital component of the stomatognathic system, serving not only in the initiation and aid of digestion but also in speech articulation and aesthetics. However, dental implants have emerged as the newest therapeutic option, offering a more predictable course of treatment and long-lasting results compared to traditional restorations [1]. Research has shown that implants designed based on Brånemark principles result in effective long-term recovery. [3] Subsequent biological and biomechanical research has further enhanced the functionality of titanium implants, [3] leading to predictable soft and hard tissue integration [4]. The advancement of implant-based therapy has been one of the most significant discoveries in dentistry over the last 40 years [5] driven by improvements in implant morphology and a deeper understanding of the treatment's biological basis. The concept of immediate loading, where implants are loaded with prosthetics shortly after placement, is based on the idea of restoring proper form and function quickly. While early attempts at immediate loading faced challenges due to a lack of understanding of biological and mechanical concepts, advancements in implant science and materials have led to improved success rates [6]. In the anterior aesthetic zone, immediate loading can improve the patient's experience during treatment by minimizing the number of surgical visits, shortening the length of treatment, and providing instant aesthetic effects [7]. Therefore, it is of interest to document the Immediate and conventional loading of anterior dental implants for aesthetic considerations and outcomes.

## Material and Methods:

## Study Design and Setting:

A randomized controlled trial was conducted at NIMS Dental College and Hospital, Rajasthan, from June 2022 to February 2024, involved 30 patients with missing anterior teeth. Sample size was determined using G Power software (version 3.1.9.7) with effect size=0.3, α error=0.02, and power=0.90. Ethical approval was obtained from NIMS University (Reference: NIMSUR/IEC/2022/282, Proposal: IEC/P-09/2022), and all procedures adhered to ethical standards and the Helsinki Declaration of 1975, as revised in 2000. The study was performed in the Department of Oral and Maxillofacial Surgery.

## Inclusion criteria:

Patients aged 18-50 years with single or multiple missing anterior teeth, healthy edentulous sites, adequate bone volume, good oral hygiene, good systemic health, and availability for follow-ups.

## Exclusion criteria:

Patients with insufficient bone quantity or quality and those with medically compromised conditions, such as smoking, TMJ disorders, bruxism, parafunctional habits, site infections, pregnancy or lactation, and those who have undergone head and neck radiation therapy, may face additional challenges in their treatment and recovery.

## Study procedure:

A pre-designed questionnaire collected data on age, gender, medical history, and comprehensive evaluations. Patients were randomized into:

## Group A (Immediate Loading):

15 patients received provisional crowns within 48 hours of implant placement.

## Group B (Conventional Loading):

15 patients had implants unloaded and restored after a 3-6month healing period. Both groups underwent a 12-month follow-up to assess bone volume reorganization.

## Initial evaluation:

Patient preparation involves educating and motivating patients to maintain optimal oral hygiene. Phase I periodontal therapy was administered, focusing on addressing underlying periodontal issues. Patients demonstrating good oral health and had plaque and gingival index values that were less than 20% after therapy were taken into consideration. Before the procedure, a thorough examination was conducted, which included complete blood counts, studying of diagnostic cast, photographs, and standardized intra oral periapical radiographs. Radiographic and clinical evaluations of the accessible bone were used to determine the implant's length and diameter. Prior to participating in the study, all patients were told about the protocol and gave their consent.

## Surgical procedure:

Patients received antibiotics one day before surgery. Local anaesthesia was administered, and a full-thickness mucoperiosteal flap was raised. Osteotomy sites were prepared per manufacturer instructions, with thorough irrigation and monitoring. Adin threaded root form dental implants were placed with 45 N torques. Postoperative care included analgesics, antibiotics, and chlorhexidine gluconate rinses.

## Prosthetic procedure:

## Group A:

Implants were immediately loaded with provisional crowns. Impressions were taken with addition silicone material, and provisional restorations were made with die stone and secured with Type I GIC. Final PFM restorations were placed after three months.

## Group B:

After three months, a second-phase surgery exposed the implant, removed excess tissue, and placed a healing abutment. Soft tissue contouring and closed tray impressions were performed. Final prosthetic parts were cemented using glass-ionomer cement.

## Radiological assessment:

Bone quantity at implant sites was assessed using intra-oral periapical radiographs and the long cone paralleling technique. Evaluations were conducted post-implantation, and at 6 and 12 months, with digitization and analysis using OMS Imaging software. Measurements included crestal bone levels, with three readings averaged for bone loss determination.

## Soft tissue evaluation:

Peri-implant soft tissue evaluation was conducted at two time period after implant placement. One at the 6-month mark referred to as Soft Tissue Time 1 (ST1), and another at 12- month referred as Soft Tissue Time 2 (ST2). Using William's graded probe and a mouth mirror, the principle worker and co-worker evaluated the soft tissue. To assess the soft tissues around the implant Plaque index by Mombelli *et al.* the Gingival Index by Apse *et al.* Calculus Index and Papilla preservation Index by Daniele *et al.* were utilized.

## Outcome assessment:

Data was collected at baseline, as well as at 6-month and 12-month intervals following randomization. The primary outcome was to determine whether there are differences in marginal bone levels between immediate loading and conventional loading of dental implants. The secondary outcomes included assessing periodontal conditions and the preservation of interdental papillae to evaluate aesthetic results between the two loading methods.

## Statistical analysis:

All the data of baseline and recall examination will be entered into Microsoft Office Excel and transferred into SPSS Software for further statistical analysis. Mean and Standard deviation was calculated. Unpaired t-Test was applied for intergroup difference and paired t-Test was applied for intragroup difference. P value <0.05 will be considered statistically significant.

## Results:

A total of 30 patients with missing anterior teeth were treated in this study. Each missing teeth was considered as an individual sample. There were 17 males and 13 females included in the study. The age ranged from 18 years to 50 years, with a mean age of 35.4 years. The recordings were noted post-operatively at 6-month and 12-mark. The mean peri-implant bone loss on the mesial side for both immediate and conventional loading of implants at the end of 6th month was 0.75mm and 0.85mm which was not statistically significant and at the end of 12th month was 1.46mm and 1.52mm (p value 0.620) which was also not significant. (Table 1) The mean peri-implant bone loss on the distal side for both immediate and conventional loading of implants at the end of 6th month was 0.78mm and 0.80mm which was not statistically significant and at the end of 12th month was 1.54mm and 1.56mm (p value 0.917) which was also not significant. (Table 2) The mean plaque score for both immediate and conventional loading of implants at the end of 6th month was 1.73 and 1.13 which was statistically significant but at the end of 12th month the plaque score was 2.33 and 2.00 (p value 0.228) which showed not significant disparity. (Table 3) The mean gingival score for both immediate and conventional loading of implants at the end of 6th month was 1.20 and 1.07 which was statistically not significant and at the end of 12th month was 1.93 and 1.93 (p value 1.000) which showed not significant. (Table 4) The mean calculus score for both immediate and conventional loading of implants at the end of 6th month was 0.60 and 0.40 which was statistically not significant and at the end of 12th month was 0.87 and 0.73 (p value 0.379) which showed not significant. (Table 5) The mean papilla preservation score for both immediate and conventional loading of implant at the end of 6th month was 2.53 and 2.33 which was statistically not significant and at the end of 12th month was 1.93 and 2.00 (p value 0.790) which showed not significant (Table 6).

## Discussion:

Implant dentistry has evolved beyond focusing solely on achieving high implant survival rates through conventional loading methods. Patients and healthcare providers now seek not only successful osseointegration but also quicker restoration of mastication, phonetics, and aesthetics that implants offer. Waiting several months for osseointegration before placing the restoration can deter patients from accepting implant therapy. To address this, various loading protocols have emerged, categorized as conventional (3-6 months), early (approximately 6 weeks), or immediate (within 48 hours of implant placement) [8-9]. The objective of conventional loading (CL) of dental implants is to maintain stability during the healing phase [10]. Advancements in implant designs, surface configurations, and surgical techniques have resulted in shorter timeframes between implant placement and functional loading. However, concerns about micro movements and implant failure have historically been associated with early loading protocols. Early and immediate loading techniques are becoming increasingly favored by patients due to reduced treatment durations and minimal discomfort caused by tooth loss. However, conflicting research findings exist regarding changes in marginal bone levels when comparing conventionally loaded implants to immediately loaded ones [11].

Several studies have explored peri-implant bone alterations between conventionally and immediately loaded implants, with some reporting comparable outcomes while others noted slight differences, particularly in the mandible [12]. The data from this study suggests a slightly lower amount of peri-implant bone loss in implants which are loaded immediately (Group A) to the one loaded conventionally (Group B) at end of 6-month mark post- implant placement. In particular, Group A exhibited an average bone loss of 0.77mm, while Group B showed 0.83mm of bone loss. Despite a difference of 0.06mm in loss of bone among both groups, there was no significance statistically. Guruprasada *et al.* suggested that peri-implant bone loss in Group A could have been impacted by factors like trauma by surgery and micro-movements by implant due to functional and non-functional stress from the tongue and lip [13]. Despite the lack of functional loading for 6 months in Group B patients, non-functional forces from the tongue and lip could have applied pressure on the implant abutment. This scenario might have led to heightened bone loss due to disuse atrophy resulting from the absence of stimulation of peri-implant tissues.

After one year of implant placement, the average annual crestal bone loss was 1.50mm for implants in immediate loading (Group A) and 1.54mm for implants in conventional loading (Group B). Both values fall beneath the 1.5mm annual loss of bone threshold in the initial year of implant services, meeting the criteria of success by Albrektsson. While the implants which are loaded immediately (Group A) displayed lower loss of bone compared to the conventional (Group B), the disparity between both groups lacked statistical significance. Results align with findings from other studies conducted by various authors [14, 15, 16]. For example, Crespi R conducted a study comparing crestal level of bone changes surrounding single implant placed in extraction socket within maxilla's aesthetic area, between implants loaded immediately and conventionally. Outcomes suggested that rate of success and radiographic results of implants restorated immediately in extraction socket were similar to the group of conventional loading [17]. Similarly, Ebenezer V reported that most immediately placed implants demonstrated excellent osseointegration [18].

Immediate loading scenarios typically exhibit a progressive type of bone resorption from baseline to subsequent intervals after implant placement. Mechanical strain induced by occlusal stresses triggers immediate bone remodeling, stimulating osteoblasts for bone deposition over time following loading [19]. However, delayed-loading shows rapid bone-resorption within initial weeks following implant surgery [20]. This attributed to active bone remodeling approximately 8 weeks into the healing process [21]. The initial bone loss observed during healing is mitigated by preventing bone remodeling and the formation of a biological seal around the implant to protect the healing site. At second phase surgery, bone typically displays lower density and weaker characteristics compared to 6-12 months post-prosthetic loading. Woven bone replaces devitalized bone in response to surgical trauma, with complete mineralization and organization enhancing bone strength over time. Occlusal stress induces micro fractures or overload in woven bone initially, but complete mineralization and organization enhance bone strength over time, enabling it to withstand similar levels of stress. The study's findings demonstrated positive clinical outcomes for both immediate and conventional loading protocols, including favorable implant survival rates, success, and aesthetic integration. Immediate loading offers shorter treatment timelines and enhanced patient satisfaction with early prosthetic restoration. However, concerns about increased peri-implant bone loss with immediate loading have been raised.

Assessment of soft tissue using various indices provided valuable insights into the periodontal status of both loading protocols. Maintaining excellent oral hygiene before implant surgery is paramount for achieving successful osseointegration and ensuring implant maintenance. The mean periodontal status of both immediate and conventional loading groups did not show statistically significant differences, emphasizing the importance of comprehensive oral hygiene management. Aesthetic outcomes and patient satisfaction are essential considerations in implant dentistry, particularly in the anterior region. Both immediate and conventionally loaded implants achieved favourable aesthetic results, with high rates of patient satisfaction reported in both groups. Achieving harmonious gingival contours and natural-looking prosthetic restorations requires close collaboration between clinicians, prosthodontists, and dental technicians.

## Limitations:

The study's limitations include a small sample size of 30 patients, which may not provide broad generalizability or account for individual variability in healing responses. The one-year follow-up period might be insufficient to capture long-term outcomes or late complications related to implant loading. Patient adherence to oral hygiene and follow-up care could also influence results, while differences in surgical techniques and operator skills might affect the reproducibility of findings.

## Conclusion:

The comparison of immediate and conventional loading protocols for anterior implants reveals similar outcomes in peri-implant bone loss. Immediate loading may cause progressive bone resorption due to occlusal stresses, while delayed loading can lead to initial rapid bone loss, though differences are not statistically significant. Patient-specific factors like bone quality and occlusal forces are crucial in determining the optimal loading protocol. Ongoing research is needed to refine these protocols and improve patient outcomes. This study adds valuable insights to guide clinicians in choosing the best loading strategy for anterior implants.

## Figures and Tables

**Table 1:** Comparison of Mean value of mesial crestal bone loss

**Evaluation in months**	**Group**	**N**	**Mean**	**Std.deviation**	**P value**
6 months	A	15	0.754	0.17455	0.200(N.S)
T1 - T0(mesial)	B	15	0.848	0.21525	
12 months	A	15	1.4593	0.30351	0.620(N.S)
T2 - T0(mesial)	B	15	1.5193	0.3494	
N.S. = not significant
* = significant (p value <0.05)

**Table 2:** Comparison of Mean value of distal crestal bone loss

**Evaluation in months**	**Group**	**N**	**Mean**	**Std.deviation**	**P value**
6 months	A	15	0.784	0.20687	0.857(N.S)
T1 - T0(distal)	B	15	0.802	0.32298	
12 months	A	15	1.5427	0.44837	0.917 (N.S)
T1 - T0(distal)	B	15	1.558	0.34364	
N.S. = not significant
* = significant (p value <0.05)

**Table 3:** Comparison of Mean value of plaque score

**Evaluation in months**	**Group**	**N**	**Mean**	**Std.deviation**	**P value**
P.I.(6 months)	A	15	1.73	0.884	0.042*
	B	15	1.13	0.64	
P.I.(12 months)	A	15	2.33	0.724	0.228[N.S.]
	B	15	2	0.756	
N.S. = not significant
* = significant (p value <0.05)

**Table 4:** Comparison of Mean value of gingival score

**Evaluation in months**	**Group**	**N**	**Mean**	**Std.deviation**	**P value**
G.I.(6 months)	A	15	1.2	0.862	0.646[N.S]
	B	15	1.07	0.704	
G.I.(12 months)	A	15	1.93	0.799	1.000[N.S.]
	B	15	1.93	0.799	
N.S. = not significant
* = significant (p value <0.05)

**Table 5:** Comparison of Mean value of Calculus score

**Evaluation in months**	**Group**	**N**	**Mean**	**Std.deviation**	**P value**
C.I.(6 months)	A	15	0.6	0.507	0.289[N.S]
	B	15	0.4	0.507	
C.I.(12 months)	A	15	0.87	0.799	0.379[N.S.]
	B	15	0.73	0.799	
N.S. = not significant
* = significant (p value <0.05)

**Table 6:** Comparison of Mean value of Papilla preservation scores

**Evaluation in months**	**Group**	**N**	**Mean**	**Std.deviation**	**P value**
P.I.I.(6 months)	A	15	2.53	0.516	0.285[N.S]
	B	15	2.33	0.488	
P.I.I.(12 months)	A	15	1.93	0.594	0.790[N.S.]
	B	15	2	0.756	
N.S. = not significant
* = significant (p value <0.05)
